# 非小细胞肺癌EGFR-TKIs耐药——小细胞肺癌转化的研究进展

**DOI:** 10.3779/j.issn.1009-3419.2017.10.10

**Published:** 2017-10-20

**Authors:** 文秋 张, 永琦 李, 荻 吴

**Affiliations:** 130021 长春，吉林大学第一附属医院肿瘤中心 Department of Tumor Centre, the First Afliated Hospital of Jilin University, Changchun 130021, China

**Keywords:** 肺肿瘤, 表皮生长因子受体酪氨酸酶抑制剂, 耐药, 机制, 转化, Lung neoplasms, Epidermal growth factor receptor tyrosine kinase inhibitor, Drug resistance, Mechanism, Transformation

## Abstract

表皮生长因子受体酪氨酸激酶抑制剂（epidermal growth factor receptor tyrosine kinase inhibitors, EGFR TKIs）在治疗携带*EGFR*基因敏感突变的非小细胞肺癌（non-small cell lung cancer, NSCLC）中已取得显著疗效，但是，耐药的产生几乎是不可避免的，常见的耐药机制包括T790M突变、*cMET*基因扩增等。目前已有文献报道EGFR-TKI耐药的机制之一为NSCLC转化为小细胞肺癌（small cell lung cancer, SCLC），大约占3%-15%，是一种重要的少见耐药机制，并不为人们所深入了解。本文从“共同起源”和“转化时间节点”两个角度对其进行了归纳总结，重点探讨了其转化的可能机制，目前提出的两种可能转化机制分别为肿瘤异质性假说、NSCLC转化为SCLC假说，还涉及了许多分子水平的改变，如*RB1*基因缺失、*P53*基因失活、*PTEN* M264I基因突变等，同时对该种转化的发病特点、治疗策略等方面进行了归纳与总结。目前仍有许多问题需要进一步研究和解决。

## Abstract (en)

The use of epidermal growth factor receptor tyrosine kinase inhibitors (EGFR-TKIs) in the treatment of sensitive *EGFR* mutation in non-small cell lung cancer (NSCLC) has been proved significant curative effect. However, the acquisition of the drug resistance to EGFR-TKIs is almost inevitable, and common drug resistance mechanisms include T790M mutation, cMET amplification, *etc*. One of the rare resistance mechanisms of EGFR-TKIs is the transformation from NSCLC into small cell lung cancer (SCLC), which account for about 3%-15%. It is an important rare drug resistance mechanism which is not well understood. Therefore, it is necessary to review the present situation and the progress of the this drug resistance mechanism. This article summarizes these hypothesizes from two parts, which are respectively the "common origin" and "transformation time node". At present, two possible mechanisms of this kind of transformation has been proposed, which are respectively the hypothesis of the tumor heterogeneity and the hypothesis of the transformation from NSCLC into SCLC. This article also involves a lot of changes in the level of molecules, such as the lack of *RB1* gene, the inactivation of *P53* gene and the mutation of *PTEN* M264I gene, *etc*. At the same time, this article summarizes the characteristics, the diagnostic methods and the treatment strategy of this kind of transformation. There are still many problems which need further research and resolution.

近年来，由于分子靶向药物对携带敏感突变基因的癌细胞存在精准性和敏感性，分子靶向治疗已逐渐在非小细胞肺癌（non-small cell lung cancer, NSCLC）的治疗中占据重要地位，现已成为NSCLC的一线治疗方案^[1, 2]^。其中在使用表皮生长因子受体酪氨酸酶激酶抑制剂（epidermal growth factor receptor tyrosine kinase inhibitors, EGFR-TKIs）治疗携带*EGFR*基因敏感突变的患者中，相比传统的化疗方案，不仅改善了患者的无进展生存期，而且提高了患者的生活质量^[3-6]^。但是，EGFR-TKI耐药的产生几乎是不可避免的，中位无进展生存期仅为11个月。2006年，Zakowski等^[7]^首次发现了经过EGFR-TKI治疗后NSCLC转化为小细胞肺癌（small cell lung cancer, SCLC）继而出现耐药的案例。2013年，NSCLC的NCCN指南明确指出EGFR-TKI的耐药机制之一为NSCLC转化为SCLC，大约占3%-15%的比例^[8-10]^。相比经典SCLC，转化的SCLC主要发生在携带*EGFR*基因敏感突变的不吸烟的亚洲女性腺癌患者^[11-13]^。

## 1 NSCLC与SCLC细胞肿瘤干细胞的共同起源

肿瘤干细胞（cancer stem cells, CSCs）是指存在于肿瘤组织中的具有干细胞性质的一小部分细胞群体，具有自我更新、分化潜能及高致瘤性和耐药性的特点。

目前主流观点认为NSCLC与SCLC细胞来源于共同的肿瘤干细胞，即两者具有“共同起源”。Tatematsu等^[14]^报道了携带*EGFR*敏感突变肿瘤细胞的起源细胞具有分化为神经内分泌肿瘤细胞的潜能，如分化为SCLC细胞。临床前证据表明，在靶向干扰*TP53*基因和*RB1*基因的条件下，肺泡Ⅱ型细胞也具有转化为SCLC细胞的潜能^[15]^。此外，肺泡Ⅱ型细胞还可作为携带*EGFR*基因突变的腺癌细胞的起源细胞^[16, 17]^。因其同时具有转化为NSCLC细胞和SCLC细胞的潜能，故肺泡Ⅱ型细胞可视为腺癌和SCLC的肿瘤干细胞^[18]^。EGFR在肺泡Ⅱ型细胞中具有重要作用^[19, 20]^，分化良好的肺泡Ⅱ型细胞EGFR高表达^[21]^。Miettinen等^[20, 22]^认为，*EGFR*基因突变和活跃的EGFR信号共同驱动肺泡Ⅱ型细胞的增殖和分化。当EGFR信号被EGFR-TKI抑制时，肺泡Ⅱ型细胞可以发生其他关键基因事件，如*RB1*基因缺失，促进其在独立于EGFR信号的情况下转化为SCLC，并产生对EGFR-TKI的耐药；然而，若肺泡Ⅱ型细泡发生其他耐药突变，如*EGFR* T790M突变，EGFR信号可以促使肺泡Ⅱ型细胞保留腺癌组织学成分^[19]^。

## 2 NSCLC EGFR-TKIs耐药-SCLC转化发生机制的几种假说

NSCLC发生EGFR-TKIs耐药转化为SCLC的发生机制仍不清楚。目前，针对该机制学者们共提出了两种假说，分别为肿瘤异质性假说、NSCLC转化为SCLC假说。

### 2.1 肿瘤异质性假说

肿瘤异质性是恶性肿瘤的特征之一。肿瘤异质性是由癌细胞中遗传学和表观遗传学差异造成的，这些因素共同作用下，导致了带有不同表型的疾病现象^[23]^。

由于肿瘤异质性，在EGFR-TKI治疗前，肿瘤组织中可能同时存在NSCLC和SCLC细胞两种成分，但病理组织学活检只找到NSCLC成分，在EGFR-TKI对NSCLC起效的过程中，SCLC的成分逐渐显现出来，并占据主导地位^[24]^。

目前针对这种假说尚有争议。支持者认为穿刺活检标本仅取材部分肿瘤组织，基于部分组织的病理诊断具有局限性，不能全面反映整体肿瘤组织的情况，有NSCLC和SCLC细胞两种成分同时存在的可能性。Mangum等^[25]^报道，同时含有NSCLC和SCLC细胞成分的异质性肿瘤在穿刺活检标本及手术切除标本中，分别占1%-3.2%、9%-26%的比例。由于EGFR-TKI治疗后二次活检的病理组织学和基因检测结果绝大多数是通过穿刺活检标本而非手术切除标本获得，因此这些病理学综合信息受到限制，由此可能造成抽样误差^[26]^。然而，反对者认为，这种假说是错误的。EGFR-TKI对携带*EGFR*敏感突变的NSCLC治疗有效且疗效显著，患者无进展生存期（progression-free survival, PFS）可达1年甚至数年。SCLC的生物学行为特点为进展迅速、临床症状明显，若肿瘤早期就含有SCLC成分，在治疗过程中将会出现明显的临床症状如刺激性咳嗽、呼吸困难等，并发生早期转移^[9]^。

### 2.2 NSCLC转化为SCLC假说

在肿瘤干细胞增殖分化的某个阶段，肿瘤干细胞先向NSCLC细胞定向分化，在NSCLC细胞增殖发展的过程中，接受某些外界压力，如EGFR-TKI，转化为SCLC。有一些学者支持该观点。Sequist等^[7, 8, 10, 27, 28]^认为，由于转化的SCLC细胞仍然携带原有*EGFR*基因敏感突变，故可以证明SCLC并非新发^[9]^，而是由原有NSCLC细胞转化而来。*EGFR*基因突变既可以存在于NSCLC细胞，又可以存在于SCLC细胞，进一步提示了含有*EGFR*基因突变的肿瘤干细胞的存在，该肿瘤干细胞可能为EGFR-TKI耐药的起源^[29, 30]^。

既往研究表明，EGFR-TKI并不是诱导NSCLC向SCLC转化的唯一因素。NSCLC转化为SCLC的发生可以独立于针对EGFR的抑制^[10]^。1986年的一项统计数据显示，5%的患者最初病理组织学诊断为NSCLC，化放疗耐药后再次行活检，病理组织学诊断为SCLC^[31]^。Watanabe等^[32]^选取手术切除标本进行病理组织学诊断，发现6例最初诊断为腺癌的患者发生了SCLC转化，其中仅有2例患者接受过EGFR-TKI的治疗，1例患者未接受任何治疗。

除此之外，SCLC转化的发生还可以独立于*EGFR*基因状态。Sequist等^[8]^对接受放化疗后的NSCLC手术切除标本进行研究，发现EGFR野生型NSCLC比*EGFR*突变型NSCLC发生SCLC转化的可能性小。

根据以上研究可以推测，EGFR-TKI及*EGFR*基因状态均不是促进SCLC转化的独立因素，但均可以作为促进SCLC转化的诱发因素，即携带*EGFR*敏感基因突变的NSCLC本身具有转化为SCLC的潜质，在EGFR-TKI的暴露环境中，更易转化为SCLC。

### 2.3 两种假说之间的联系与区别

上述两种假说之间具有一定关联。根据转化时间节点不同，可将转化的机制分为“同时性假说”和“异时性假说”，即“肿瘤异质性假说”和“NSCLC转化为SCLC假说”。前者发生的转化时间节点为使用EGFR-TKI治疗之前，后者发生的转化时间节点为使用EGFR-TKI治疗之后（图 1）。

**1:**
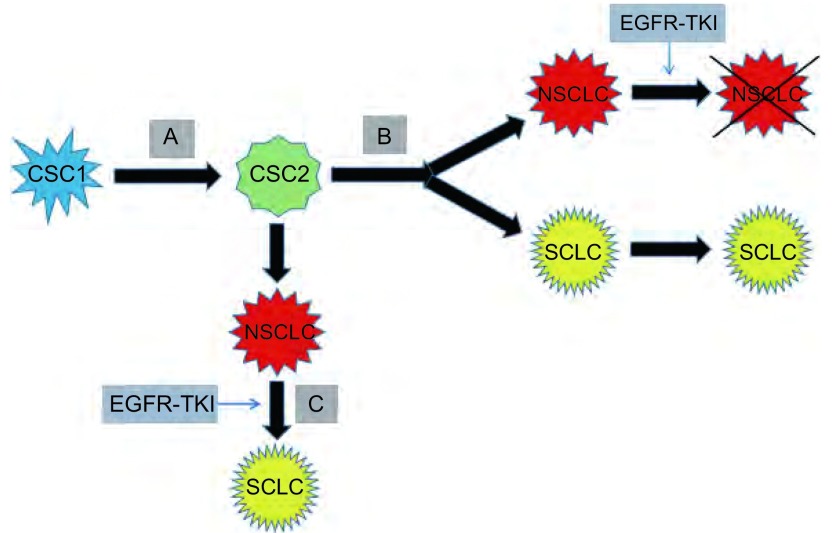
“共同起源”与两种假说之间的联系与区别。A：共同起源；B：肿瘤异质性假说；C：NSCLC转化为SCLC假说。肿瘤干细胞（CSC1）在增殖分化的过程中，分化为具有特定分化能力的肿瘤定向干细胞（CSC2），该肿瘤定向干细胞既可能同时分化为NSCLC细胞和SCLC细胞，形成异质性肿瘤，EGFR-TKI可减少或消灭NSCLC细胞，而SCLC细胞残留并逐渐占主导作用，即“肿瘤异质性假说”，也可能先分化为NSCLC细胞，该NSCLC细胞在EGFR-TKI的压力下转化为SCLC细胞，即“NSCLC转化为SCLC假说”。 The association and difference between the origin of cancer stem cell and two hypothesis. A: The origin of cancer stem cell; B: The hypothesis of the tumor heterogeneity; C: The hypothesis of the transformation from non-small cell lung cancer (NSCLC) to small cell lung cancer (SCLC). During the process of proliferation and differentiation, an original cancer stem cell (CSC1) differentiate into an cancer stem cell (CSC2) with the ability of specific differentiation. This cancer stem cell (CSC2) can not only differentiate into NSCLC and SCLC cell and form the tumor heterogeneity at the same time, and EGFR-TKI can reduce or eliminate NSCLC cells, and SCLC cells residue and gradually play a leading role, which is the hypothesis of the tumor heterogeneity, but also differentiate into NSCLC cell firstly, then transform into SCLC under the pressure of EGFR-TKI, which is the hypothesis of the transformation from NSCLC into SCLC.

## 3 NSCLC EGFR-TKIs耐药-SCLC转化的分子机制

虽然越来越多的SCLC转化作为EGFR-TKI耐药机制的案例被报道出来，但其分子机制仍然未知。许多研究已发现一些与SCLC转化相关甚至起到重要作用的分子事件。

### 3.1 *RB1*基因缺失

*RB1*基因在细胞周期G_1_期起调控作用，*RB1*基因的缺失会造成细胞周期G_1_期失控，在SCLC转化中起重要作用。经典SCLC 100%存在*RB1*基因缺失。高频率的*RB1*基因缺失发生于SCLC、前列腺癌、膀胱癌^[33]^。后两者都经历了小细胞分化^[34, 35]^。

动物实验证明*RB1*基因的缺失选择性影响神经内分泌起源的肿瘤，导致原发神经内分泌肿瘤细胞数量的增加^[36]^。分子测序证实，*EGFR*突变耐药转化的SCLC细胞，100%存在*RB1*基因缺失。SCLC转化前的NSCLC及SCLC转化后仍存在的NSCLC成分均不存在这种基因改变，提示*RB1*基因缺失在SCLC转化中扮演重要角色^[22]^。

研究发现单独敲除*RB1*基因并未引起*EGFR*基因突变细胞向神经内分泌方向分化，证明SCLC转化不仅仅依赖于*RB1*基因的缺失^[22]^。同时，*RB1*基因缺失的腺癌细胞的存在，进一步证明了仅有*RB1*基因的缺失对SCLC转化是不够的^[37]^。

### 3.2 *P53*基因失活

*P53*基因为抑癌基因，若*P53*基因失活，将失去其对细胞生长、凋亡和DNA修复的正常调控作用，对肿瘤形成起重要作用。

研究证明靶向干涉神经内分泌细胞的*P53*基因和*RB1*基因会导致SCLC的发生^[38, 39]^，靶向断裂肺泡Ⅱ型细胞的*P53*基因和*RB1*基因也会导致SCLC的发生^[15]^，这不仅进一步证明了肺泡Ⅱ型细胞可作为SCLC的起源细胞，而且证明了*P53*基因和*RB1*基因在SCLC转化中扮演着重要角色。

### 3.3 *PTEN* M264I基因突变

*PTEN*基因为抑癌基因，作用为负性调控PI3K-AKT途径，起到促进凋亡和抗增殖的作用^[40]^。在研究肺腺癌经吉非替尼治疗后发生SCLC转化并转移至肝脏的案例中，发生SCLC转化的肿瘤组织中发现*PTEN* M264I基因突变，仍为腺癌成分的肿瘤组织中未发现该基因突变^[41]^，此突变在腺癌中极少见^[42-44]^，据此可以推测其在SCLC转化中具有一定作用。

### 3.4 *PIK3CA*基因突变

PI3K/AKT信号通路是重要的抗凋亡通路，而且与新生血管的生成有关。EGFR与其配体结合后，引起自身磷酸化和二聚体化，激活PI3K/AKT下游信号通路，并产生一系列磷酸化效应，这些效应蛋白共同促进肿瘤细胞的生长和蛋白质合成。

Sequist曾报道SCLC转化伴有*PIK3CA*基因突变的案例，提示PIK3-AKT通路的激活可能参与SCLC的转化。然而，*PIK3CA*基因的激活突变还出现在大约5%的NSCLC患者中^[45]^。所以，对SCLC转化的发生而言，仅有该通路的激活是不够的。

## 4 转化的SCLC和经典SCLC之间的相似性

转化的SCLC与经典SCLC在基因、形态学、临床表现、药物敏感性等方面具有相似性^[22]^。

基因上，RNA和微小RNAs表达的等级聚类分析表明，转化的SCLC与经典SCLC相似，免疫组织化学染色显示神经内分泌标记物如突触小泡蛋白、嗜铬粒蛋白、神经元粘附分子阳性表达^[8, 10, 24, 34, 46, 47]^；形态学上，两者均表现为小细胞；临床表现上，SCLC进展迅速、易早期转移，转化后的SCLC增殖率快速增加、临床表现快速恶化，转移较早；药物敏感性上，两者均对标准SCLC化疗方案敏感^[22]^。

虽然上述几个方面体现了转化SCLC与经典SCLC之间的相似性，然而，已发现一组经典表达在NSCLC中的微小RNA及一组经典存在于NSCLC的甲基化DNA可表达及存在于一些转化的SCLC。因此转化的SCLC并不能被归为经典SCLC，也许可被归为一种新的SCLC亚型^[22]^。

## 5 NSCLC EGFR-TKIs耐药-SCLC转化的治疗对策

转化SCLC与经典SCLC具有以上多方面的相似性，可以给予“耐药机制为基础”的治疗。使用标准SCLC化疗方案“顺铂/卡铂+依托泊苷”治疗转化SCLC，疗效大多显著^[8]^，但有效治疗过后，由于转化SCLC具有较强侵袭性以及患者体力状况（performance status, PS）评分等因素，大多数患者的病情在初期得到缓解后迅速进展直至患者死亡。研究数据表明，相比经典SCLC，转化SCLC的预后相对较差。而且，SCLC转化在EGFR-TKI难治的转移性肿瘤中占重要部分^[41]^。

除此之外，由于携带*EGFR*敏感突变的SCLC对EGFR-TKI治疗有效，因此可推测NSCLC转化的SCLC可能对EGFR-TKI同样有效^[9, 48]^，但较NSCLC疗效差。Sequist等^[8]^报道称，转化为SCLC的患者在接受EGFR-TKI与化疗治疗的过程中，肿瘤对EGRR-TKI可以经历“耐药-重新敏感-耐药”的改变，同时伴随基因和表型改变（图 2）。该案例表明，没有持续的EGFR-TKI选择性压力，耐药机制所涉及的基因和表型改变会发生丢失，这些肿瘤对第二轮EGFR-TKI治疗敏感有效。在不同药物治疗的选择性压力之下，会出现不同克隆的选择性^[22]^。同时，该案例还提示了肿瘤对EGFR-TKI耐药具有潜在的可逆转性，进一步强调了一次活检不足以指导后续治疗，再次活检甚至三次活检具有重要指导意义^[8]^。

**2:**
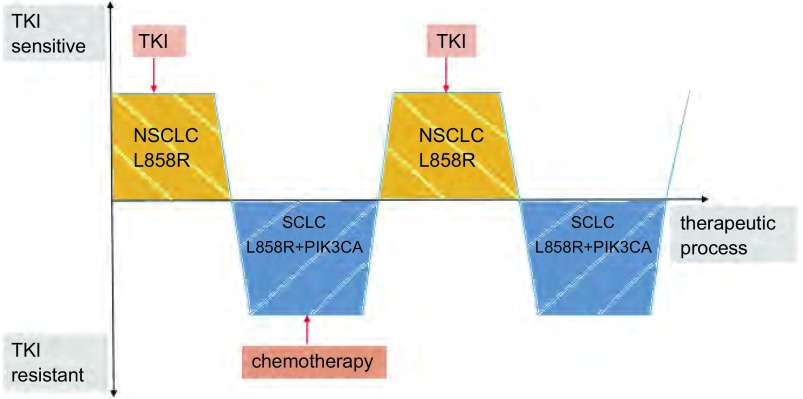
肿瘤对EGFR-TKI敏感或耐药及其基因和表型变化。携带*EGFR* L858R敏感突变的NSCLC经历EGFR-TKI治疗后转化为SCLC，转化SCLC保留原有*EGFR* L858R突变，同时携带新发PIC3CA突变，经过7个月的化疗，再次活检，发现SCLC重新转化为NSCLC，该NSCLC只携带原有*EGFR* L858R敏感突变，新发PIC3CA突变消失，此时继续给予EGFR-TKI，再次因SCLC转化的发生产生耐药，此SCLC同样保留原有*EGFR* L858R基因突变，同时含有新发PIC3CA突变。 The change of the sensitivity or resistance and gene and phenotype of tumor after the treatment of EGFR-TKI. NSCLC which had sensitive *EGFR* L858R mutation transformed into SCLC after the treatment of EGFR-TKI, and the transformed SCLC not only retained the original *EGFR* L858R mutation, but also contained new *PIC3CA* mutation. After 7 months of chemotherapy, the second biopsy revealed NSCLC, which only contained sensitive *EGFR* L858R mutation, and no new *PIC3CA* mutation was found. At this time, EGFR-TKI was given again, then the resistance occured again because of SCLC transformation, which not only retained the original *EGFR* L858R mutation, but also contained new *PIC3CA* mutation.

Shoemaker等^[49]^称，在临床前研究中，navitoclax（ABT-263），一种BCL-2、BCL-XL抑制剂，在治疗经典SCLC中显示出显著的疗效。尽管近期临床试验结果证明单药ABT-263仅在少部分患者中取得疗效^[50]^，但联合用药方案仍在探索中^[51]^。Niederst Sequist等^[22]^试验报道称，携带*EGFR*基因敏感突变NSCLC转化的SCLC细胞系不仅对ABT-263高度敏感，促使其产生强效凋亡作用，而且敏感度明显强于T790M基因突变耐药的NSCLC细胞系。这些结果表明含有ABT-263的联合方案在转化SCLC的治疗中非常具有潜力。

Kenichi ^[41]^称，在EGFR-TKI耐药过程中，肿瘤可能同时含有多个EGFR-TKI难治性耐药机制，如T790M二次突变和SCLC转化共同存在于同一个患者。因此，在EGFR-TKI耐药的后续治疗中，考虑到一个患者体内可能有不同耐药机制以及肿瘤存在内在异质性是重要的。

## 6 研究的局限性及遗留问题

目前，尽管对于SCLC转化作为EGFR-TKI耐药机制之一的研究已取得较大进展，但是仍有一些需要进一步研究和探讨的问题。

由于SCLC转化的发生，对SCLC进行重新分型是必要的。转化SCLC也许可以被归为SCLC的一个亚型，即源于肿瘤干细胞分化或NSCLC转化并且含有*EGFR*基因敏感突变的一类SCLC，其无标准治疗方案，预后差，总生存期短。

值得注意的是，发生SCLC转化的大部分患者为不吸烟女性，这提示性别因素比如激素水平或者激素受体水平的不同是否在揭示SCLC转化的过程中起到线索作用^[41]^?

尽管*EGFR*基因敏感突变持续存在，但转化为SCLC的患者对EGFR-TKI出现耐药，这可能是由于EGFR在蛋白水平的表达缺失^[8]^。未来也许可以根据检测EGFR蛋白表达水平决定是否继续使用EGFR-TKI^[52]^。Chang等^[53]^还提出SCLC转化可能仅为耐药的伴随现象，而非主要原因的猜想。

## 7 小结

本文综述了SCLC转化作为NSCLC对EGFR-TKI的耐药机制之一的研究进展及耐药后治疗的策略，虽然仍然有许多需要进一步研究和解决的问题，但已有的研究结果为进一步研究提供了有利的线索，耐药机制的深入研究提供了新的治疗路线，不仅有助于指导临床用药，而且将促进更有针对性的靶向药物的研发。对以上假说进行验证性研究，进而明确其转化的分子机制，收集大量病例资料进行全面的前瞻性研究，包括生物学特性、对目前治疗措施的应答率、预后等，是研究者们未来需要解决的问题。
